# Clinical and Laboratory Profile of Zika and Dengue Infected Patients: Lessons Learned From the Co-circulation of Dengue, Zika and Chikungunya in Brazil

**DOI:** 10.1371/currents.outbreaks.0bf6aeb4d30824de63c4d5d745b217f5

**Published:** 2018-02-15

**Authors:** Elzinandes Leal Azeredo, Flavia Barreto dos Santos, Luciana Santos Barbosa, Thiara Manuele Alves Souza, Jessica Badolato-Corrêa, Juan Camilo Sánchez-Arcila, Priscila Conrado Guerra Nunes, Luzia Maria de-Oliveira-Pinto, Ana Maria de Filippis, Márcia Dal Fabbro, Izilyanne Hoscher Romanholi, Rivaldo Venancio da Cunha

**Affiliations:** Viral Immunology Laboratory, Oswaldo Cruz Institute, Rio de Janeiro, Brazil; Viral Immunology Laboratory, Oswaldo Cruz Institute, Rio de Janeiro, Brazil; Viral Immunology Laboratory, Oswaldo Cruz Institute, Rio de Janeiro, Brazil; UFRJ- Federal University of Rio de Janeiro, Laboratory of Genetics, IPPMG - Martagão Gesteira Child Care and Pediatrics Institute, Rio de Janeiro, Brazil; Viral Immunology Laboratory, Oswaldo Cruz Institute, Rio de Janeiro, Brazil; Viral Immunology Laboratory, Oswaldo Cruz Institute, Rio de Janeiro, Brazil; Laboratory of Viral Immunology, Fundação Instituto Oswaldo Cruz, Rio de Janeiro, Brazil; Viral Immunology Laboratory, Oswaldo Cruz Institute, Rio de Janeiro, Brazil; Fundação Oswaldo Cruz, Research, Immunology, Rio de Janeiro, Brasil; Flavivirus Laboratory, Oswaldo Cruz Institute, Rio de Janeiro, Brazil; Medical Clinic Department, Federal University of Mato Grosso do Sul, Campo,Grande, MS, Brazil; Faculdade de Medicina, Universidade Federal de Mato Grosso do Sul, Campo Grande, Mato Grosso do Sul, Brasil; Medical Clinic Department, Federal University of Mato Grosso do Sul, Campo,Grande, MS, Brazil

## Abstract

Background: The current triple epidemic caused by dengue, zika and chikungunya constitutes a serious health problem in Brazil. The aim of this study was to investigate acute samples (up to the 7 days of symptoms) from patients presenting acute fever syndrome suspected as arboviral infection and characterize the clinical and laboratorial profile during the co-circulation of dengue, zika and chikungunya in Campo Grande, Mato Grosso do Sul (MS), midwest region of Brazil. Methods: All suspected cases (n=134) were tested by using serological and molecular diagnostic tests including DENV, ZIKV and CHIKV RT-PCR, Dengue nonstructural protein 1 (NS1) antigen capture ELISA, anti- DENV IgM ELISA and anti-CHIKV IgM ELISA. In addition, clinical, hematological and biochemical parameters of infected patients were analyzed. Results: It was observed that 79.1% of the blood samples were confirmed for ZIKV and/or DENV infection Of those, 38.0% patients were DENV monoinfected, 26.8% were ZIKV monoinfected and 13.4% were DENV/ZIKV co-infected. Seven patients presented Chikungunya IgM antibodies indicating a previous CHIKV infection. Common symptoms included fever, rash, arthralgia, myalgia, prostration, headache and conjunctivitis. Statistical analysis showed that pruritus and edema were associated with ZIKV infection while prostration and vomiting were more associated with dengue. Additionally, total protein and ALT levels were significantly different in DENV patients compared to ZIKV ones. Some DENV infected patients as well as co-infected needed hospitalization and venous hydration. Otherwise, most ZIKV infected patients presented mild clinical course. Among the pregnant women studied (n=11), three were ZIKV monoinfected while four were DENV monoinfected and two were DENV-1/ZIKV coinfected. In general, normal birth outcomes were observed except for the death due to respiratory insufficiency of one baby born to a mother coinfected with DENV-1/ZIKV. Conclusions: Herein, we provide evidence of the co-circulation of DENV, ZIKV and CHIKV infections in the Campo Grande, MS, Brazil, with a high frequency of DENV-1/ZIKV coinfection. Laboratorial diagnosis poses a challenge where those arboviruses are endemic and differential diagnosis proved to imperative for cases characterization. The knowledge about disease severity during arbovirus coinfections is still scarce and there are several issues emphasizing the importance of adequate management of patients at risk areas.

## Introduction

Dengue (DENV), Chikungunya (CHIKV) and Zika (ZIKV) viruses have been receiving a lot of attention, especially due to the recent high number of cases of chikungunya and zika in Brazil^1^.

DENV and ZIKV, single-stranded positive RNA viruses, belong to the *Flaviviridae* family and *Flavivirus* genus. Dengue, an acute disease caused by any of the four serotypes (DENV-1 to 4) may range from an acute undifferentiated febrile illness to a more severe form, characterized by bleeding and plasma leakage 2. ZIKV was first isolated from a sentinel rhesus monkey in Ziika forest, Uganda^3^ and the infection may result in asymptomatic to symptomatic manifestations. Although zika symptoms are generally mild, severe neurological complications have been reported including Guillain-Barré syndrome (SGB) and microcephaly, mainly in regions with DENV circulation^4^. CHIKV belongs to the Togaviridae family and Alphavirus genus, and cause an acute febrile disease characterized by severe and debilitating arthralgia^5^.

For the past 30 years, the Brazilian population has been suffering the consequences of dengue epidemics and, since DENV-1 introduction in the 80's^6^, the number of severe cases increases, changing the disease epidemiology and concerning the public health authorities. The recent introduction of CHIKV and ZIKV in Brazil, reinforces the need for a better understanding of the co-circulation of more than one arboviruses in the same environment, as well as their impact in the susceptible population.

In 2015, Brazil reported 1,65 million dengue suspected cases and 854 deaths due to the disease. After its emergence in 2015, ZIKV spread in the country and a total of 165,932 suspected cases were reported in 2016^7^.

In MS, Midwest region of Brazil, a total of 1,801 zika suspected cases were reported in 2016 and, due to the high vector density and susceptible individuals, the risk for a chikungunya outbreak in Brazil was eminent. In that year, a total of 71,824 chikungunya suspected cases were reported, with 15.1318 cases and 196 deaths laboratorially confirmed^7^. In this study, we analyzed patients presenting acute fever syndrome suspected of arboviral infection during the 2016 outbreak in city of Campo Grande, MS, Brazil and the clinical, laboratorial and virological aspects are discussed.

## Methods

**Ethics Statement**

The cases analyzed in this study were from an ongoing Project approved by the Oswaldo Cruz Foundation Ethic Committee (CAAE 57221416.0.1001.5248). All patients enrolled signed a written consent. The patients data were collected by an infectious disease physician using a structured questionnaire. The patients personal information was anonymized before the data was accessed. This study accessed the patients information on demographic characteristics, physical signs and symptoms.

**Study population**

This is a cross-sectional and observational study carried out in Brazil during DENV and ZIKV epidemic occurred in 2016. Patients were assisted at the Healthy Unit UPA Coronel Antonino in Campo Grande, MS, Brazil from February to March of 2016. During the study 134 suspected arboviral infection cases were included and submitted to investigation. Patients presenting fever, rash during acute phase of infection (up to the 7th day after disease onset) followed by at least two of the following signals and symptoms: headache, myalgia or arthralgia, conjunctivitis, pruritus, retro-orbital pain and prostration were recruited as suspicion of arboviral infection. Moreover, patients with acute onset of generalized macular or papular rash, pruritus and conjuctival hyperemia were considered as zika suspected cases according to the Brazilian Ministry of Healthy Protocol^8^. Patients with acute fever presenting two or more of the following signs or symptoms: nausea, vomiting, myalgia, arthralgia, headache, retro orbital pain, petechiae, positive tourniquet test, thrombocytopenia and leucopenia were considered as dengue suspected cases according to the Brazilian Ministry of Health^9^.

An infectious disease physician collected data on demographic characteristics, symptoms and physical signs. Detailed clinical examination was registered immediately after admission and blood was routinely collected for complete blood counts, liver enzymes dosage, total proteins and albumin determination. Acute serum samples (up to the 7th day after disease onset) were stored at -70°C until processing.

Dengue cases were classified according to the 2009 WHO criteria^10^, as follows: Dengue without warning signs (DwoWS): patients living in and/or traveling to dengue endemic area, presenting fever and two of the following symptoms: nausea, vomiting, rash, pain, positive tourniquet test and leukopenia; Dengue with warning signs (DwWS): dengue patients with any of the following warning signs: abdominal pain or tenderness, persistent vomiting, clinical fluid accumulation, mucosal bleeding, lethargy or restlessness, liver enlargement >2 cm, and an increase in hematocrit concurrent with rapid decrease in platelet count; Severe dengue (SD): dengue patients presenting at least one of the following: severe plasma leakage (leading to shock and fluid accumulation with respiratory distress), severe bleeding evaluated by clinicians, severe involvement of liver by aspartate aminotransferase (AST) or alanine transferase (ALT)>1,000U, central nervous system with impaired consciousness, and severe involvement of the heart and other organs (WHO/TDR, 2009). Zika cases were classified according to the 2016 Brazilian Ministry of Healthy Protocol^8^ and the protocol described elsewhere^11^.

**Laboratorial diagnosis**

All cases were screened for DENV, ZIKV and CHIKV as a differential diagnosis. For dengue serological diagnosis, suspected cases were submitted to the Dengue Virus IgM Capture DxSelect ™ (Focus Diagnostics, California, USA) and Platelia ™ Dengue NS1 Ag ELISA ELISA (BioRad Laboratories, California, USA). Molecular detection and serotype typing were performed by conventional RT-PCR as described previously^12^ and by the real-time RT-PCR^13^. The viral RNA was extracted using the QIAamp Viral RNA Mini kit (Qiagen, Germany) following the manufacturer’s instructions and stored at -70°C. The specific IgG antibody analysis was performed to determine whether the dengue infection was either primary or secondary as described previously^14^. Aiming to further exclude DENV infection in negative cases, all samples were further tested by using the Simplexa™ Dengue Real Time RT-PCR (Focus Diagnostics, Cypress, CA) according to the manufacturer´s protocol, for viral qualitative detection and typing of DENV. Due to the cross-reactivity among flaviviruses in the serological methods, suspected zika cases were tested by the real-time RT-PCR for ZIKV, as described previously^15^. Chikungunya infection in acute samples was determined by using the RT-PCR protocol described elsewhere^16^. The determination of anti-CHIKV IgM antibodies was performed according to the in-house IgM capture ELISA described by the CDC and the Ministry of Health (2014) and by anti-CHIKV ELISA IgM (Euroimmun, Lubeck, Germany)^17^ according to the manufacturer's protocol. All samples were also subjected to a RT-PCR for detection of alphaviruses (Mayaro virus – MAYV) according to de Morais Bronzoni et al^18^.

**Statistical analysis**

Statistical analyses were performed using GraphPad Prism software, version 6.0 (GraphPad Software Inc., San Diego, CA, USA). The nonparametric Mann–Whitney U test was used to evaluate differences between groups DENV and ZIKV. Fisher test was used to evaluate frequencies of positivity in sign or symptoms. Values of p < 0.05 were considered significant for all statistical analysis.

## Results

**Study population**

**Laboratorial diagnosis of arbovirus infection**

In this investigation, 134 suspected cases of arboviral infection were analyzed at the acute phase of infection. A total of 107 patients with positive diagnosis of dengue or zika infections in the acute phase [median (95% CI): 4 (3.5-5.2) during the 2016 epidemic in MS, Brazil were analyzed. The mean age was 34 years old and 46 were female and 32 male (46F: 32M). Demographical, clinical and laboratorial data from the different patient groups enrolled in this study are shown in Table 1.

**Table 1:** Demographic characteristics of the population and laboratorial parameters of studied patients during the triple epidemic in Campo Grande, MS, Brazil, 2016.

	ZIKV^δ^	DENV	CHIKV	NEGATIVE^ϕ^
	(n = 38)	n = 69)	(n = 7)	(n = 28)
Age	31 (30.5-40.1)	32 (32.7-40.3)	26 (17.5-53.8)	38 (32.1-44.3)
Gender				
Female: Male (n)	21:17	32:37	2:5	22:6
Pregnant (n)	5	5	0	1
Post-infection days^a^	3.5 (3-4.1)	4.0 (4.1- 6.2)	3.0 (1.9 – 4.9)	3.0 (1.3- 5.7)
Hospitalization, n (%)	2 (0.9)	7 (5.22)	1 (0.9)	0
Comorbities				
Arterial hypertension, n (%)	8 (7.5)	12 (11.5)	2 (1.8)	1 (0.94)
Diabetes mellitus, n (%)	0	2 (1.8)	2 (1.8)	0
Other comorbidities^♯^, n (%)	9 (8.4)	19 (17.9)	1 (0.9)	8 (7.5)

^δ^ Total positive for arbovirus infection regardless monoinfetion or coinfection; ^ϕ^ Patients that were negative for all methodologies applied; ^a^ Days from disease onset until the interview; ^♯^ Other comorbidities include rhinitis, bronchial asthma, sinusitis and rheumatoid arthritis;Data are presented as medians (interquartile range) or numbers (n) with percentage (%).

Seventy-nine percent (107/134, 79%) of the cases were confirmed by using any of the diagnostic laboratorial performed. Thirty patients (30/134, 22.3%) presented DENV IgM antibodies as evaluated by MAC-ELISA. Anti-DENV NS1 was detected in 50 out 134 (37.3%) of the cases. In addition, 7 (7/134, 5.22%) patients had specific CHIKV IgM antibodies. The RT-PCR assays for DENV, ZIKV and CHIKV were used for virus screening. A total of 69 (69/134, 51.4%) samples were positive for DENV and 38 (38/134, 28.3%) for ZIKV (Table 2).

Aiming to exclude DENV infection in negative cases, all samples were also tested by the Simplexa TM Dengue Real Time RT-PCR and protocol described by Johnson et al (2005). DENV-1 (89.8. %; 62 out 69) and DENV-4 (10.1%; 7 out 69) were the infecting serotypes detected. DENV RNA monoinfection was detected in 51 tested samples (51/134,38.1%); DENV and ZIKV RNA were detected simultaneously in 18 (18/134,13.4%). ZIKV RNA monoinfection were detected in 18 cases (18 out 134, 13.4%), which were negative by Dengue NS1 ELISA and Dengue IgM ELISA. Two ZIKV PCR positive samples were also positive for dengue NS1. No tested samples were positive for CHIKV RNA by RT-PCR. DENV-1 was the predominant infecting serotype among DENV monoinfected patients and co-infected ones (45/51 [88.2%] of monoinfections and 17/18 [94.4%] of confection), followed by DENV-4 (6/51 [11.7%] of monoinfections and 1/18 [5.5%] of coinfections), Table 2.

**Table 2:** Laboratorial diagnosis of arboviruses suspected cases (n=134) analyzed from the triple outbreak occurred in 2016 in Campo Grande, MS, Brazil.

Laboratorial diagnostic methods	Positive/Tested (%)
Dengue IgM ELISA	30 (22.3)
Dengue NS1 ELISA	50 (37.3)
CHIKV IgM ELISA	7 (5.6)
DENV RT-PCR ^£^	69 (51.4)
DENV-1	62 (89.8)
DENV-4	7 (10.1)
ZIKV RT-PCR ^£^	38 (28.3)
CHIKV RT-PCR	0
Mono and co-infections	
DENV monoinfection^¥^	51 (38.0)
ZIKV monoinfection^ω^	18 (13.4)
DENV/ZIKV coinfection ^$ω^	18 (13.4)

^£^ Protocols performed: conventional RT-PCR (Lanciotti et al, 1992), Simplexa^TM^ Dengue Real Time RT-PCR and Real Time RT-PCR (Johnson et al, 2005). All positive samples for DENV or ZIKV RT-PCR despite coinfection or presence of CHIKV anti- IgM antibodies; ^¥^ Serotypes of DENV monoinfection: DENV-1 (45/51 [88.2%]) and DENV-4 (6/51 [11.7%]); ^$^ Serotypes of DENV/ZIKV coinfection: DENV-1 (17/18 [94.4%]) and DENV-4 (1/18 [5.5%]); ^ω^ Since we considered only molecular diagnosis for mono and co infections classification, two ZIKV PCR positive patients who also presented dengue NS1 positive test were not include in the analyses as ZIKV monoinfection or DENV/ZIKV coinfection.

The Ct (Cycle Threshold) values by RT-PCR observed for DENV monoinfected cases ranged from 22.3 to 38.5, for ZIKV from 31.3 to 38.2 and DENV/ZIKV coinfections from 25.4 to 38.5 and 31.3 to 37.5 respectively (Figure 1). Lower Ct values indicate higher viral burden. Although we did not observe statistical difference between the different groups analyzed, co-infected DENV/ZIKV cases tended to present lower Ct values than those observed for DENV or ZIKV monoinfected.

**Real time RT-PCR Ct (Cycle Threshold) values observed for (A) DENV, (B) ZIKV and (C) DENV/ZIKV coinfected patients in MS, Brazil, 2016.**
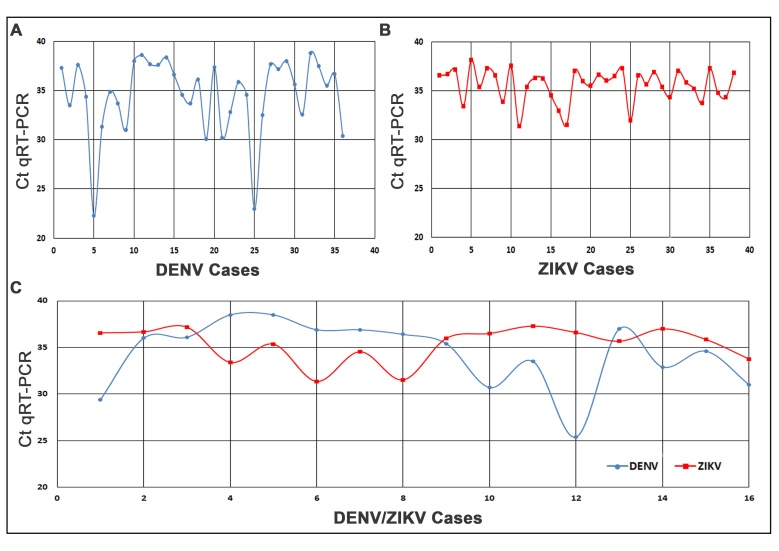

Sero-surveillance of the patients showed that 12 (12/51; 23.5%) of the DENV RNA monoinfected patients were positive for anti-DENV IgM while DENV/ZIKV coinfected presented three cases positive for anti-DENV IgM (3/18, 16.6%). Only one ZIKV monoinfected patient (1/ 18, 5.5%) was positive for anti-DENV IgM.

In addition, ZIKV (n=1) and DENV-1 (n=3) patients by RT-PCR (4/134, 2.9%) presented specific anti-CHIKV IgM antibodies. Three (3/134, 2.2%) negative cases for DENV and ZIKV RT-PCR that were negative for anti-DENV IgM and Dengue NS1 tests, presented specific anti-CHIKV IgM antibodies. Moreover, DENV PCR positive cases were significantly more likely to be positive for DENV NS1 antigen capture test than ZIKV PCR positive (39/51 versus 1/18, p=0.0001). Similarly, DENV/ZIKV PCR positive samples were significantly more likely to be positive for NS1 test than ZIKV positive ones (8/18 versus 1/18, p=0.0178).

Since MAYV belongs to the alphavirus group along with CHIKV and it is known to circulate in Brazil, all samples were tested by MAYV RT-PCR and no positive case was found. In 20.1% (27/134) of the cases, arbovirus infection was not confirmed after testing by all laboratorial methods and therefore were considered as negative.

**Clinical characteristics of DENV and ZIKV infected patients**

Since most patients in this study were positive for DENV or ZIKV infections, a comparison of signs and symptoms was performed (Table 3). The zika group was characterized by high frequency of exanthema and pruritus while the dengue was characterized by high frequency of vomiting, anorexia, prostration and dizziness. Co-infected DENV/ZIKV patients presented lower frequency of exanthema and pruritus. Statistical analysis showed that prostration and vomiting were significantly associated with DENV monoinfection (90.7%, 49/6, p=0.0311; 31.4%, 17/54, p=0.0146 respectively). On the other hand, pruritus and edema were more associated with ZIKV monoinfection (86.6%, p=0.0181; 66.7%, p=0.0172, respectively). There was a trend toward more frequent edema in women compared to men (53.3%, p=0.0617). Patients with DENV/ZIKV coinfection presented significantly less headache than patients with DENV monoinfection (p=0.0101), Table 3

**Table 3:** Signs and symptoms of zika and dengue infected patients during the 2016 epidemic, in Campo Grande, MS. Brazil.

Signs and symptoms (%)	ZIKV (n=15)	DENV^Φ^ (n=54)	DENV/ZIKV (n=15)	Negative* (n=28)
Fever	13 (86.6)	46 (85.1)	11 (73.3)	19 (67.8)
Headache	13 (86.6)	48 (88.8)	†10 (66.6)	18 (78.5)
Conjuctival hyperemia	8 (53.3)	19 (43.1)	9 (60.0)	7 (25.0)
Retro-orbital pain	14 (93.3)	43 (79.6)	11 (73.3)	15 (60.7)
Myalgia	12 (80.0)	49 (90.7)	14 (93.3)	21 (71.4)
Arthralgia	10 (66.6)	46 (85.1)	13 (86.6)	21 (75.0)
Anorexia	6 (40.0)	34 (62.9)	6 (40.0)	14 (50.0)
Prostration	10 (66.6)	^£^49 (90.7)	13 (86.6)	21 (75.0)
Dizziness	6 (40.0)	31 (57.4)	8 (53.3)	11 (39.2)
Nausea	5 (33.3)	36 (66.6)	6 (40.0)	11 (39.2)
Vomiting	0	^+^17 (31.4)	2 (13.3)	4 (14.2)
Epigastralgia	0	1 (2.1)	0	0
Abdominal pain	3 (20.0)	24 (44.4)	4 (26.6)	9 (32.1)
Adenomegaly	0	1 (1.85)	0	0
Pruritus	^£^ 13 (86.6)	28 (51.8)	7 (46.6)	21 (75.0)
Paresthesia	6 (40.0)	17 (31.4)	5 (33.3)	8 (28.5)
Exanthema	12 (80.0)	32 (59.2)	8 (53.3)	19 (67.8)
Edema	^£^6 (40.0)	6 (11.1)	5 (33.3)	9 (32.1)
Diarrhea	3 (20.0)	15 (27.7)	2 (13.3)	5 (17.8)
Cough	2 (13.3)	16 (29.6)	2 (13.3)	8 (28.5)
Low back pain	10 (66.6)	38 (70.3)	9 (60.0)	15 (64.2)
Painful hepatomegaly	0	2 (3.7)	2 (13.3)	1 (3.57)

^Φ^ 10 patients with negative RT-PCR result for DENV, ZIKV and CHIKV, but which a NS1 positive test result were considered as DENV monoinfected and included in the analyses; ^†^ p<0.05 represent statistical difference DENV vs DENV/ZIKV; ^£^ p<0.05 represent statistical difference ZIKV vs DENV or DENV/ZIKV; ^+^ p<0.05 represent statistical difference ZIKV vs DENV; ^*^ Patients that were negative for all methodologies applied.

Pregnant women and patients with a previous CHIKV IgM antibody were not included in the analyses.

Statistical significance was assessed using Fisher`s exact test (two-sided) Prism 6 Statistics (Graphpad, EUA).

Among eleven pregnant women analyzed, four were positive for DENV, three for ZIKV and two for DENV-1/ZIKV. One out of 11 (9,1%) pregnant woman was DENV, ZIKV and CHIKV RT- PCR negative, but presented anti-DENV IgM antibodies. Only one pregnant woman was negative for all methods tested. All ZIKV infected pregnant women presented anti-DENV IgG antibodies. No difference was observed in relation to clinical manifestations or laboratorial parameters between pregnant women infected with ZIKV as compared to those infected with DENV. In general, ZIKV infected pregnant women had less fever and prostration, but due to the small sample size, no statistics was performed (Table 4. Eight birth outcomes were available. As demonstrated in Table 4, fetuses of 3 out 5 ZIKV positive pregnant women analyzed had normal intrauterine ultrasonography as well as normal head circumference. The babies are healthy since birth according mother’s reports. In addition, only newborns from ZIKV/DENV-1 coinfected pregnant women presented abnormal outcomes despite normal intrauterine ultrasonography and normal head circumference as well. One of them was born with functional plagiocephaly and was in intensive care unit (ICU) for 2 months. The other baby died immediately after birth with respiratory insufficiency. All DENV infected pregnant women had normal birth outcomes and their babies are healthy.

**Table 4:** Demographic, clinical and laboratorial characteristics of pregnant women enrolled in this study.

Case	Gestacional age (weeks)	Signs and Symptoms	ZIKV molecular diagnosis	DENV molcular diagnosis	anti-DENV IgM	anti-DENV IgG	DENV NS1 anigen capture	Newborn outcome
1	27	Headache, Exanthema, Pruritus, Abdominal pain	+	-	-	+	-	Alive. Healthy child since birth
2	20	Arthralgia, Exanthema, Pruritus	+	-	+	+	+	Alive. Healthy child since birth
3	12	Headache, Prostration, Myalgia, Low back pain, Anorexia, Nausea, Vomiting	-	+	-	+	-	Alive. Healthy child since birth
4	9	Fever, Headache, Prostration, Abdominal pain, Exanthema, Pruritus, Retro-orbital pain	+	+	-	+	-	Alive. Functional plagiocephaly
5	28	Headache, Arthralgia, Low back pain, Exanthema, Pruritus	-	-	-	+	-	Not available
6	26	Fever, Prostration, Myalgia, Arthralgia, Retro-orbital pain, Anorexia, Exanthema, Pruritus	-	+	+	+	+	Alive. Healthy child since birth
7	36	Fever, Headache, Prostration, Low back pain, Retro-orbital pain, Nausea, Exanthema, Pruritus, Dizziness	-	+	-	+	-	Alive. Healthy child since birth
8	28	Fever, Headache, Prostration, Myalgia, Retro-orbital pain	-	+	-	+	-	Not available
9	16	Headache, Myalgia, Arthralgia, Low back pain Exanthema, Pruritus, Retro-orbital pain, Anorexia	+	-	-	+	+	Not available
10	20	Fever, Exanthema, Conjutival hyperemia, Retro-orbital pain	+	+	-	+	-	Deceased. Respiratory insufficiency
11	21	Headache Myalgia	-	-	+	+	+	Alive. Healthy child since birth

**Differential laboratorial characteristics during DENV and ZIKV infection**

Platelets counts and hematocrit test are used in the evaluation of dengue severity and also as a differential diagnosis. Therefore, monoinfected DENV patients presented low platelets count as compared to the negative cases (Table 5). As expected, lower platelets counts were found in DwWS/Severe DENV monoinfected patients when compared to those without WS (Supplemental Table). Nevertheless, DwWS/Severe DENV monoinfected patients presented significantly lower platelets counts as compared to DwoWS DENV/ZIKV coinfected patients (median x10^3/^mm^3^ (95% CI): 101 (39.4-172.1) and 188 (155-224), respectively). In addition, among DENV monoinfected patients, 9 out 54 (16.6%) had platelets counts ≤ 100.000/mm^3^ while all ZIKV monoinfected and coinfected ones had platelets counts ≥ 100.000/mm^3^.

DENV and ZIKV monoinfected patients showed lower leukocytes counts as compared to negative cases. Although, we observed that DENV monoinfected patients tend to present lower lymphocytes counts, only ZIKV monoinfected showed significantly lower lymphocytes counts as compared to negative ones (median x103/mm^3^ (95% CI): 1330 (990.2-2453) vs. 2035 (1650-2442) respectively). Interestingly, both monoinfected DENV or ZIKV as well as coinfected patients had a lower eosinophil counts as compared with negative cases (median x103/mm^3^ (95% CI): 50.5 (63-148); 54 (36.5-11); 44 (23.3-182.3) vs. 124 (104-208) respectively), but only ZIKV infected had counts below normal reference values (Table 5).

**Table 5:** Laboratorial characteristics of the DENV, ZIKV or DENV/ZIKV co-infected patients during the 2016 outbreak occurred in Campo Grande, MS, Brazil.

Laboratorial parameters	Negative	DENV	ZIKV	DENV/ZIKV
AST, IU/L ^a^(95% C.I.)^ a^	20 (16.9-26.9)(n=11)	36.4 (36.4-75.8) ^++^(n=28)	27.5 (21.4-32.5)(n=8)	23 (10-115.7)^+ ^(n=9)
ALT, IU/L ^a^	18 (14.1-24.4)(n=9 )	33.5 (34.9-59.6) ^++ £^(n=44)	23 (17.6-28.0) (n=13)	24 (11.7-74.7) (n=15)
Total proteins (g/dL)	7.2 (6.9-7.4)(n=18 )	7.3 (7.1-7.4)^£ ^(n=46 )	7.7 (7.3-7.9)^+ ^(n=13 )	7.4 (7.1-7.6) (n=15 )
Total albumin (g/dL)	4.2 (4.0-4.3)(n=18 )	4.0 (4.0-4.3) (n=46 )	4.2 (4.0-4.4) (n=13 )	4.3 (4.1-4.4) (n=15 )
Hematocrit (%)	39 (38.7-41.2)(n=23 )	41 (39.8-42.1) (n=54)	41 (39.4-44) (n=15 )	43 (39.2-44.2) (n=15 )
Platelets, 10^3^/mm^3^ counts ^a^	222.5 (205.5-244)(n=22)	190 (154.8-194.6)^++^, (n=54)	197 (155.5-265.3)(n=15)	193 (160.1-229.9)(n=15)
Leukocytes, mm^3^ counts	6200 (5317-7966)(n=17)	4400 (4599-6860)^+^(n=53)	4400 (4102-5178)^++ ^(n=15)	4600 (3717-7096)(n=15)
Lymphocytes, mm^3^ counts	2035 (1650-2442)(n=13)	1558 (1351-1897)(n=50)	1330 (990.2-2453) ^+^(n=15)	1136 (1012-1887)^+ ^(n=15)
Monocytes, mm^3^ counts %	520.5 (415.2-613.1)(n=14)	396 (362-514.5)(n=50)	429 (361.1-536.4)(n=15)	470 (394.1-574.1)(n=15)
Neutrophils, mm^3^ counts % ^a^	2940 (2573-4225)(n=21)	2631 (2404-4106)(n=42)	2187 (1904-3218)(n=12)	2478 (1832-4928)(n=15)
Eosinophils, mm^3^ counts % ^a^	124 (104-208)(n=21)	50.5 (63-148)^+^(n=42)	54 (36.5-111)^++^(n=12)	44 (23.3-182.3)^+^(n=15)
Basophils, mm^3^ counts % ^a^	0.0 (5.3-28)(n=21)	0.0 (11-46) (n=41)	19.5 (6.4-52.0)(n=12)	0.0 (1.9-35.8) (n=15)

^a^ Median C.I., 95% Confidence interval; ^+^ p<0.05; ^++^ p<0.01 represent statistical difference negative vs DENV, ZIKV or DENV/ZIKV.

Considering the biochemical parameters, the circulating AST and ALT levels were significantly different during DENV infection. DENV monoinfection and DENV/ZIKV coinfection presented higher circulating levels of AST compared to negative cases [median x103/mm3 (95% CI): 36.4 (36.4-75.8); 23 (10-115.7) vs. 20 (16.9-26.9) respectively]. Similarly, DENV monoinfected patients presented higher ALT levels compared to ZIKV monoinfected and to negative cases (median x103/mm3 [95% CI]: 33.5 [34.9-59.6]; 23 [17.6-28] and 18 [14.1-24.4] respectively). In addition, levels of circulating total proteins were lower in DENV monoinfected compared to ZIKV monoinfected. Although DENV monoinfected patients tended to present low albumin levels (p=0.0632) compared to DENV/ZIKV coinfected, no significant difference was observed in patients with DENV or ZIKV monoinfection or even in coinfected ones. According dengue severity, no difference was observed in DwoWS or DwWS/severe monoinfected patients in relation to the circulating levels of albumin and total proteins (Supplemental Table).

**n **corresponds to the total number; Ω ( (LCL) Lower confidence limit; (UCL) Upper confidence limit; 95% CI of median;^a^ Illness Days corresponds to the days of the start of any symptoms until the moment when the patient was interviewed;^b^ Represent statistical difference between without WS versus with WS/ severe;c Represent statistical difference between DENV with WS/severe versus DENV/ZIKV without WS and vs ZIKV monoinfection;^d^ bleeding : methorrhagia, epistaxis and gum bleeding. Mann-Whitney nonparametric test was applied. Pregnant women and patients with a previous CHIKV IgM antibody were not included in the analyses.

**Disease severity and clinical outcomes**

Based on the 54 DENV monoinfected patients, 46 were classified as DwoWS, 7 as DwWS and one as SD^10^ On the other hand, among the 15 DENV/ZIKV coinfected patients, 12 were classified as DwoWS and 3 as DwWS (Supplemental Table). The main warning signs were painful hepatomegaly, liver enlargement, mucosal bleeding and increase in HCT concurrent with rapid decrease in platelet count.

Manifestations associated with a risk of an unfavorable dengue outcome, such as plasma leakage, severe hemorrhagic manifestations (e.g. gastrointestinal) or organ impairment (renal, hepatic neurological or cardiac), were not observed in both DENV monoinfected as well as DENVZIKV coinfected ones. Seven DENV monoinfected patients and two DENV/ZIKV coinfected were hospitalized and received parenteral hydration. Only one severe DENV monoinfected patient received platelet transfusion. All patients were discharged from hospital and there were no fatal case.

Manifestations associated with an unfavorable zika outcome, as severe neurologic complications (Guillan-Barré Syndrome, transverse myelitis) were not observed in ZIKV monoinfected patients or in DENV/ZIKV co-infected ones. Only one DENV-1/ZIKV coinfected patient was hospitalized due disorientation, blurred vision and difficulty in audition. No ZIKV monoinfected patients received parenteral hydration.

## Discussion

The present study described the first cases of zika confirmed in the municipality of Campo Grande, MS. Morever, it demonstrates the co-circulation of DENV, ZIKV and CHIKV providing evidence of DENV and ZIKV co-infection in the Midwest region of Brazil.

Although serological methods are useful tool in diagnosis of dengue infection, in our study, only 23.5% (12/54) of DENV RT-PCR positive samples presented dengue specific IgM antibodies. Routinely, both an acute and convalescent samples are needed for the laboratorial diagnosis of dengue infection since seroconversion occurs 3 to 7 days following exposure. Moreover, due to the cross-reactivity observed among flaviviruses such as DENV and ZIKV and the lack of a reliable serological test, it is considered that methods for diagnosis of those viruses in the acute phase should be performed through molecular tests and dengue NS1 antigen detection.

Little is known about false-positive dengue NS1 tests and cross-reactivity with other flaviviruses. Gyurech et al^19^ observed a false-positive result for dengue NS1 in a case of ZIKV infection. In our study, we observed that DENV PCR positive patients were significantly more likely to have a positive NS1 test result than ZIKV ones. In fact, no false-positive dengue NS1 test results were found in Zika patients from French Guiana^20^, suggesting that dengue NS1 testing is reliable and should be applicable for dengue surveillance even during ZIKV outbreaks. In fact, we found that DENV-specific T cells targeting NS1 proteins were undetectable in acute ZIKV-patients (unpublished data).

DENV-1 was the predominant serotype in this study. The high number of DENV cases in Campo Grande (MS) corroborates the data published by the Brazilian Health Surveillance Secretariat - SVS / MS^7^, which reported in MS, a total of 4,088 dengue cases in 2016. Due co-circulation of DENV, CHIKV and ZIKV in Brazil, differential diagnosis with molecular methods is essential for confirming arbovirus infections. In our study, the detection and typing of DENV in all samples was performed using more than one RT-PCR protocols^12^^,^^13^^,^^18^. This strategy improved DENV detection and a higher rate (50.0%) of co-infection by DENV and ZIKV was observed. In fact, cases of co-infections have been reported with patients with detectable RNA from two or three viruses concomitantly. The co-circulation of arboviruses resulted in the occurrence of DENV and CHIKV co-infections as observed during an outbreak in 2013 in Laos^21^ and in Thailand in 1962-1964^22^. In 2006, co-infections with DENV and CHIKV was reported in Kinta District (Asia)^23^ . More recently, ZIKV, DENV and CHIKV infections were reported in Nicaragua and coinfections with one, two or more viruses were found^24^. In addition, two CHIKV and ZIKV co-infected patients were found in state of Bahia 25, and two ZIKV and DENV co-infected patients in Pernambuco, Brazil^26^. It is likely that co-infections are not being identified and misdiagnosed since molecular testing for all arbovirus are not usually performed in the Brazilian laboratories. Most ZIKV suspected cases are not routinely confirmed by molecular tests, as during epidemics, cases investigations are restricted to pregnant women and newborns.

Clinical manifestations and laboratorial parameters were compared among patients with ZIKV, DENV or coinfected by those viruses. In general, arboviruses share similar signs and symptoms. ZIKV infected patients typically presented symptoms such as fever, rash, arthralgia, myalgia, fatigue, headache and conjunctival hyperemia in agreement with previous studies in Brazil^26^^,^^27^^,^^28^ Recently, it was demonstrated that pruritus was the most common clinical sign presented by Brazilian ZIKV infected patients^27^. According to the data obtained in the present study, we found that ZIKV monoinfected patients were more likely to have pruritus compared to DENV monoinfected ones, confirming previous observations. Interestingly, DENV monoinfected patients were more likely to present prostration and vomiting as well.

We also evaluated the laboratorial parameters as an attempt to contribute to the differential diagnosis. It is well known that dengue infections are associated with thrombocytopenia and is more pronounced in severe cases^29^. On the other hand, severe thrombocytopenia during ZIKV infection has been rarely reported^30^. In fact, we observed that only DENV monoinfected patients presented low platelets count. As described previously, in general, ZIKV patients presented moderately decrease in leucocytes and lymphocytes counts^30^. Interestingly, we found that both DENV and ZIKV infected patients tended to present low eosinophil count, but only ZIKV infected had counts below normal values. Eosinophils are associated with allergies and parasitic infection and are important mediators of innate response against pathogens. Besides, during acute infection a dramatic fall in the number of circulating eosinophils occurs^31^. MMastocytes and basophils are activated and release histamine upon stimulation by eosinophil-derived cationic granule proteins^32^. In fact, anti histamines are used to help and reduce pruritic rash during ZIKV infection^4^. However, the role of eosinophils during ZIKV or DENV infections remains to be elucidated.

ZIKV is an emerging arbovirus and therefore is yet not well described. On the other hand, the clinical manifestations of DENV are known. DENV infection can range from a nonspecific febrile illness to a more severe illness with bleeding tendency, thrombocytopenia, transaminases elevations and plasma leakage resulting in complications and death^33^. Laboratorial parameters such as platelet count^34^, urea, creatinine^35^^,^^36^, AST, ALT^37^, protein and albumin levels^35^ are described as potential indicators of ICU in dengue infection. Differentiate dengue from other arboviruses is crucial to clinicians in endemic areas since an early diagnosis allows monitoring of potential markers of dengue severity. Importantly, increases in ALT levels and decreases in protein levels were found in most DENV patients as compared to the ZIKV infected ones, suggesting that those factors should be considered as potential indicators of DENV infection. However, more prospective studies with larger sample sizes are needed to confirm these observations.

Another important aspect of this study was to evaluate whether subsequent infections or co-infections by these viruses could affect the clinical course of the disease and lead to an unfavorable outcome. According to the WHO dengue classification^10^, the diagnosis of severe dengue was not frequent in both DENV monoinfected and DENV/ZIKV co-infected. Most patients analyzed here recovered after a mild clinical course. No infected ZIKV patient had neurological manifestation and only one co-infected was hospitalized due disorientation and blurred vision but who was discharged without complications. The reports of DENV and ZIKV or even CHIKV coinfections are very limited and most studies did not find severe clinical outcomes^24^^,^^38 ^, in agreement with our data. Nevertheless, seven dengue fatal cases coinfected with CHIKV were reported in Colombia^39^. More recently, Brito et al^40^ reported ZIKV and CHIKV coinfection cases that evolved with severe neurological manifestations,. Importantly, DENV-1 and DENV-4 were the serotypes detected, and DENV-2 and DENV-3, serotypes frequently associated with a more severe, were not identified. Although, most co-infected patients presented less bleeding and high platelets counts, two co-infected patients needed venous hydration reinforcing the importance of adequate management of patients in the early recognition of the dengue warning signs. Considering the few co-infected cases reported, is not possible to ensure that co-infected individuals do not have an increased risk of dengue severity. The impact of coinfection in disease severity is unknown requiring further investigations.

Both DENV and ZIKV infected pregnant women presented similar clinical manifestations and no significant differences were found probable due to the small cohort analyzed. No abnormal birth outcomes were found among the ZIKV infected pregnant women or DENV infected ones. Importantly, DENV-1 and ZIKV coinfected pregnant women (9 weeks and 20 weeks, respectively), presented abnormal birth outcomes including functional plagiocephaly and one newborn loss due respiratory insufficiency despite normal intrauterine ultrasonography and normal head circumference. Although the greatest risk for adverse outcomes is in the first trimester, abnormal outcomes have been reported within all trimesters^41^^,^^42^^,^^43^. Our data emphasize the importance of the differential diagnosis for DENV, ZIKV and CHIKV in suspected cases regardless diagnosed ZIKV infection. The impact of DENV/ZIKV co-infection is unknown, as far as we know, and only one study reported arbovirus co-infection during pregnancy with no complications or abnormalities^9^. More importantly, it was demonstrated increased dengue severity during pregnancy emphasizing the importance of DENV infection recognition since infected pregnant woman are more disposed to develop severe dengue than non-pregnant women^44^.

Serological evidence of CHIKV infection was reported in 2.2% of the samples tested suggesting a CHIKV epidemic progression in this region in agreement with previous published data by Secretariat of Health Surveillance-SVS /MS, who registered 57 CHIKV cases in Campo Grande, until 52^nd^ epidemiological week of 2015^7^.

Although we did not analyze the viral loads and the viruses from co-infected cases were not sequenced, different molecular protocols were performed for DENV detection confirming the data accuracy. In our analysis, no significant difference was found between DENV/ZIKV co-infected Ct values and DENV or ZIKV monoinfected ones, although coinfected cases presented slightly lower Ct values than those ZIKV monoinfected. Prospective ZIKV and DENV viral loads assessment in different phases of infection as well as the evaluation of the immune response should be done in future studies. In conclusion, our data characterized the occurrence of an ongoing triple epidemic caused by DENV, ZIKV and CHIKV in Campo Grande, MS, Brazil. Importantly, the knowledge about dengue or zika severity in co-infected individuals is still unknown, however we provided here, new contributions related to the clinical presentations and laboratorial parameters during DENV and ZIKV infections, in an endemic scenario.

## Author Contributions

Patient recruitment and enrollment: RVC, ELA, TMAS, LSB, JBC, MDF, IHR; Patient Classification: RVC, ELA; Performed the experiments: ELA, TMAS, LSB, JCSA, PCGN; Contributed with reagents and lab support: ELA, FBS, RVC, LMOP, AMBF; Discussion of results: ELA, RVC, FBS, LMOP and Revised and commented on the manuscript: RVC, FBS.

## Corresponding Author

Elzinandes Leal de Azeredo (elzinandes@ioc.fiocruz.br or naideazeredo@gmail.com).

## Conflict of Interest

The authors declare that there is no conflict of interest.

## Data Availability Statement

The data analyzed is not available due ethical restrictions and confidentiality terms. Oswaldo Cruz Foundation Ethic Committee (CAAE 57221416.0.1001.5248).

## Supporting Information

**Supplemental Table:** Demographic and laboratory characteristics of suspected cases according to the type of infection (mono or co-infection) during the triple epidemic occurred in Campo Grande, MS, Brazil during 2016.

	DENV		DENV/ZIKV		ZIKV
Demographic and laboratory characteristics	Without WS (n=46)	With WS/Severe (n=8)	Without WS (n=12)	With WS/Severe (n=3)	(n=15)
Gender F:M	22:24	6:2	5:7	1:2	12:3
Age Median (LCL-UCL)	33 (31.1-39.8)	46.5 (31.15-39.8)	25.5 (23.4-44.1)	21 (12-71)	40 (32.6-45.26)
^a^ Days of disease ^Ω^Median (LCL-UCL)	3.5 (2.5-4.4)	4.5 (0.7-11)	25.5 (23.4-44.1)	21 (12-71)	40 (32.6-45.26)
DENV-1 (n/total)	33/38	5/6	12/13	3/3	0
DENV-4 (n/total)	4/38	1/6	1/13	0/3	0
Hospitalization (%)	0	1/6	0	0/3	0
AST (IU/L)(n=total)	33 (36.6-78) (n=24)	43 (18.9-69)(n=4)	23 (24-147) (n=7)	22.5 (16-28)(n=2)	27.5 (21.4-32.5) (n=8)
ALT (IU/L)(n=total)	39.7 (37-46.4)(n=41)	62.5 (113-346)(n=4)	28 (5.8-93) (n=11)	16 (10-22.5)(n=3)	23 (17-28.1)(n=13)
Total proteins (g/dL)	7.3 (7.1-7.4)(n=40)	7.0 (6.4-7.7)(n=6)	7.3 (7.1-7.4)(n=14)	7.0 (6.4-7.7)(n=3)	7.0 (6.4-7.7)(n=15)
Albumin (g/dL)	4.05 (4.07-4.2(n=40)	4.2 (3.4-4.5)(n=6)	4.3 (4.1-4.4)(n=14)	4.1 (2.4-6.0)(n=3)	4.2 (4.05-4.4)(n=13)
Hematocrit (%)	41 (40.7-42.5)(n=46)	39.5 (33.9-43)(n=8)	43 (39-44.6)(n=14)	39 (26-53.8)(n=3)	41 (39.4-44.1)(n=15)
Platelets 10^3^ /mm^3^ counts	198.5 (168-206.6) (n=46)	101 (39.4-172.1) ^b***^(n=8)	188 (147-224)^ c*^(n=12)	193 (40-392)(n=3)	197 (155.5-265.3)^ c*^(n=15)
Bleeding (%)	0	6 (75%)^d^	1 (7.14%)^b^	2 (66%)	0
